# The normalised Sentinel-1 Global Backscatter Model, mapping Earth’s land surface with C-band microwaves

**DOI:** 10.1038/s41597-021-01059-7

**Published:** 2021-10-28

**Authors:** Bernhard Bauer-Marschallinger, Senmao Cao, Claudio Navacchi, Vahid Freeman, Felix Reuß, Dirk Geudtner, Björn Rommen, Francisco Ceba Vega, Paul Snoeij, Evert Attema, Christoph Reimer, Wolfgang Wagner

**Affiliations:** 1 Technische Universität Wien, Department of Geodesy and Geoinformation, 1040 Vienna, Austria; 2 Earth Observation Data Centre for Water Resources Monitoring (EODC), 1030 Vienna, Austria; 3 Spire Global, Space Program, 2763 Sainte-Zithe, Luxembourg; 4 European Space Agency, European Space Research and Technology Centre, 2201 AZ Noordwijk, The Netherlands; 5 Airbus Defense and Space, 2333 CS Leiden, The Netherlands; 6 European Space Agency, 2200 AG Noordwijk, The Netherlands

**Keywords:** Aerospace engineering, Environmental sciences, Hydrology, Biogeography, Geomorphology

## Abstract

We present a new perspective on Earth’s land surface, providing a normalised microwave backscatter map from spaceborne Synthetic Aperture Radar (SAR) observations. The Sentinel-1 Global Backscatter Model (S1GBM) describes Earth for the period 2016–17 by the mean C-band radar cross section in VV- and VH-polarisation at a 10 m sampling. We processed 0.5 million Sentinel-1 scenes totalling 1.1 PB and performed semi-automatic quality curation and backscatter harmonisation related to orbit geometry effects. The overall mosaic quality excels (the few) existing datasets, with minimised imprinting from orbit discontinuities and successful angle normalisation in large parts of the world. Regions covered by only one or two Sentinel-1 orbits remain challenging, owing to insufficient angular variation and not yet perfect sub-swath thermal noise correction. Supporting the design and verification of upcoming radar sensors, the obtained S1GBM data potentially also serve land cover classification and determination of vegetation and soil states. Here, we demonstrate, as an example of its potential use, the mapping of permanent water bodies and evaluate against the Global Surface Water benchmark.

## Abstract (summary)

**Table:** 

Measurement(s)	C-band radar backscatter • extent of permanent water bodies
Technology Type(s)	Synthetic Aperture Radar (SAR) • Synthetic Aperture Radar (SAR) normalised mosaic
Factor Type(s)	radar backscatter
Sample Characteristic - Environment	land
Sample Characteristic - Location	global

Machine-accessible metadata file describing the reported data: 10.6084/m9.figshare.16432983

## Background & Summary

Spaceborne Synthetic Aperture Radars (SAR) scan the Earth with microwaves, supplementing our visual perceptions as captured by optical satellite missions. Analogous to optical sensors that record reflected sunlight in the visible and infrared spectrum, they enable the retrieval of geophysical variables, as e.g. vegetation density^1–3^, forest condition^4–6^, soil moisture^7–9^, snow cover^10,11^, land cover^12,13^, and water extent^14–16^. While satellite-borne optical sensors might be hampered by atmospheric elements or in case of clouds entirely blocked, microwave sensors are hardly ever disturbed by the atmosphere, due to their active radar signal transmission. They allow a clear view on the surface, under all weather conditions and at any time of day and night. High-resolution SARs, which provide a spatial resolution comparable to optical imagery, are in particular valuable when optical sensors fail. Furthermore, as they operate in the microwave spectrum and respond to different physical processes, the obtained radar signals depict an additional source of information, measuring ground variables from another physical perspective, or even reveal new properties.

The processing and interpretation of SAR data is complicated by the nature of radar signal scattering mechanisms, rendering the work with it challenging, in both engineering and scientific terms, while the radar community is lacking a robust reference dataset. A global SAR backscatter mosaic describing the Earth surface in a comprehensive and harmonised way, free from voids and artefacts, was still up to now unavailable. Albeit that SAR-satellites are orbiting since the 1970s, planetary-scale backscatter mosaics were created only recently. To our knowledge, the first global composites were created in 2010 from Envisat ASAR^17^ (500 m sampling, 5.3 GHz), and in 2013/19 from ALOS PALSAR-1/2^18^ (25 m sampling, 1.27 GHz). Although making large steps forward, those mosaics inherit inconsistencies from erratic observations patterns due to concurrent observational sensor modes.

In this regard, ESA’s 2014-launched Sentinel-1 mission^19^ represents a game changer. Employing C-band SAR instruments (CSAR) operated at a 5.5 cm wavelength, it is the first SAR mission that is dedicated to a systematic backscatter acquisition, with a two-satellite-constellation scanning global land masses at a 10 m sampling within 6 days. Owing to the mission’s unprecedented coverage, it became quickly possible to produce first global Sentinel-1 image mosaics. Examples are the composite image for 2014–17 created by Descartes Labs^20^ on the Google Earth Engine^21^, and the dual-polarisation mosaic for 2016 by the Joint Research Centre of the European Commission (JRC-EC) that demonstrated also the mapping of human settlements^22^. Notwithstanding Sentinel-1’s systematic acquisition plan, also these mosaics show some pronounced artefacts related to coverage heterogeneity, due to CSAR’s duty cycles limited by on-board power, thermal- and data-downlink -constraints, and the mission’s fixed orbit configuration that generates a discriminative swath footprint pattern^9^. Moreover, as a result of maintaining the repeat orbit acquisition geometry (that serves well SAR-interferometry), many parts of the world are observed from only one viewing angle in ascending or descending overpass direction. Especially outside Europe (which is prioritised within the duty cycles and is hence well-covered) this poses a great challenge when normalisation by (projected) local radar incidence angle (PLIA) is sought.

Not addressed so far in mosaicking endeavours, PLIA-normalisation is essential for the generation of a harmonised backscatter mosaic and for downstream applications, as radar backscatter is strongly varying with viewing angle. Here, we present the Sentinel-1 Global Backscatter Model (S1GBM), the first complete global land backscatter mosaic that is normalised to a single reference incidence angle, and hence suitable for spatially extensive analysis and application. Based on the Sentinel-1A and −1B Interferometric Wide Swath (IW) mode acquisitions^19^, it provides 2.67 TB of quality-curated layers at a 10 m sampling, and covers 97.9% of global land outside Antarctica for 2016–17. Backscatter is expressed in terms of σ° (sigma nought) at VV- and VH- polarisation, and is PLIA-normalised to an incidence angle of 38° using a linear regression method.

The S1GBM was generated to support the design, testing and verification of future C-band radar missions (Sentinel-1C/D, HydroTerra, Harmony, Sentinel-1 Next Generation (NG)), related SAR-processor performance simulations, raw data downlink compression optimisation, and for visualisation purposes. Nonetheless, it also presents a valuable environmental record for investigating the C-band VV/VH backscatter response over different land covers, and documenting the state of the land surface in 2016/17. In this publication we demonstrate the global mapping of permanent water bodies (PWB), through applying a simple threshold on the S1GBM layers. Although the algorithm is comparatively compact, results from comparison against 2015’s benchmark of Global Surface Water from JRC-EC^23^ are very encouraging. We invite developers from the broader user community to exploit this novel data resource and to integrate S1GBM parameters in models for various variables of land cover, soil composition, or vegetation structure.

## Methods

**Fig. 1:**
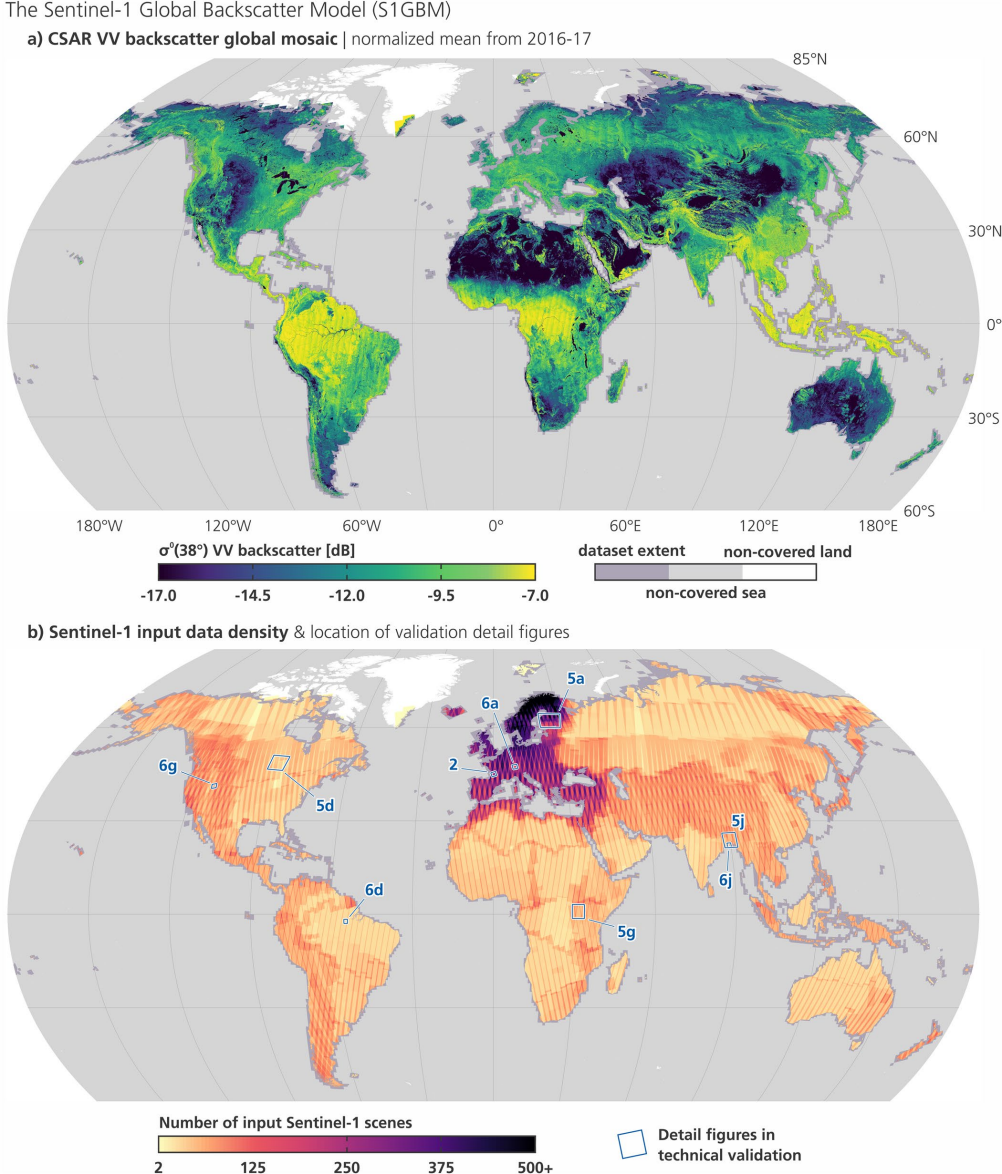
(**a**) S1GBM’s main layer, the global mosaic depicting the average VV-polarised backscatter coefficient *σ*_0_ in decibels (dB), normalised to 38° incidence angle, for the period 2016–2017. The dataset’s extent is mapped in dark grey over the sea; non-covered land areas are mapped in white (omitting non-covered Antarctica). (**b**) Input density map to the mosaic in a), mapping per pixel the number of used Sentinel-1 scenes from the period 2016–17. The S1GBM detail images displayed in the Technical Validation section are shown in blue.

**Fig. 2:**
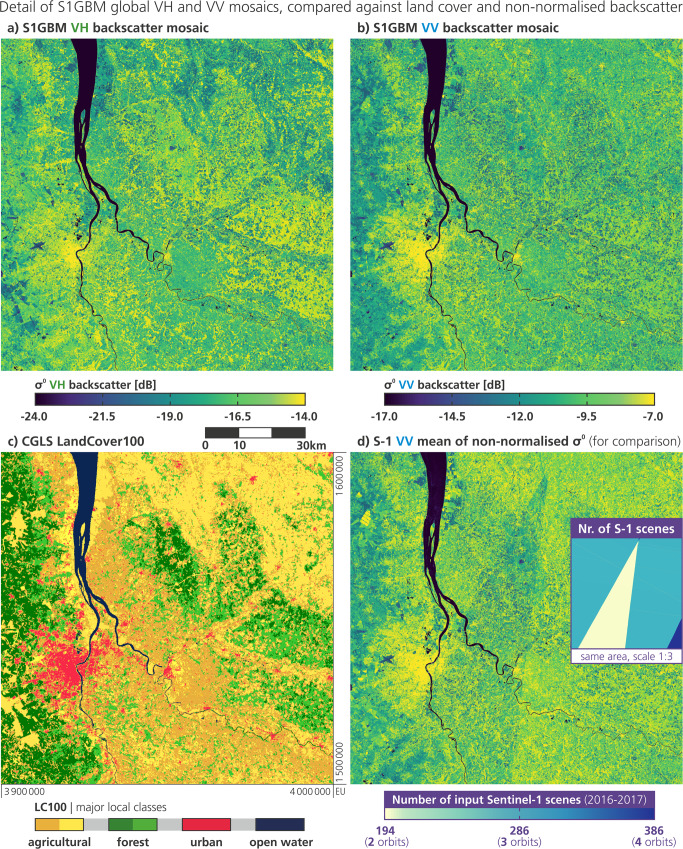
Example of the (**a**) S1GBM VH and (**b**) VV mosaics of the area of Bordeaux, France, compared with (**c**) the local Land Cover classes for the year 2015 of the Copernicus Global Land Service, and with (**d**) Sentinel-1 mean VV backscatter from the same observation period and all relative orbits covering the area, but not normalised for incidence angle (PLIA). (**d**) includes a mini-map of the same area plotting the number of Sentinel-1 observations contributing to (**b**) and (**d**) that is bound to Sentinel-1’s (heterogeneous) orbit coverage pattern.

The aim of our work was to create consistent and comprehensive mosaics from the Sentinel-1 CSAR observations over land. As a start into this paper, Fig. 1a presents the S1GBM’s main layer, the global mosaic of the normalised mean radar backscatter signal (henceforth referred to as “backscatter”) in VV polarisation covering the period 2016–17. In this overview world map, one can see the general characterisation of the Earth’s land masses as perceived by C-band radar sensors, – with high average backscatter over the tropical rain forests, – with medium backscatter in the temperate climates sustaining agrarian cultivation, – with low backscatter over the sparsely vegetated grasslands of the American Great Plains, Kazakhstan and central Asia, and the savannas of southern Africa, – and with extreme low backscatter over arid areas of the subtropics in Africa, Arabia, and Australia, as well as in China and Mongolia. Beyond those characteristics at region-scale, the mosaic holds also a variety of land-cover and geomorphologic features at field scale, delineating e.g. rivers, lakes, forests, infrastructure, and cities (compare also with the zoomed-in detail in Fig. 2a), but also surface properties bound to soil- and bedrock composition, dominating e.g. the Sahara, Arabia, Australia, and the Tibetan Plateau.

Figure 1b maps the input data density to the mosaic above, counting per mosaic pixel the number of used (and quality-checked) datasets, and reflecting to a great deal the Sentinel-1’s ground coverage pattern over landmasses of the 2016–17 period. Daily-updated global Sentinel-1 accumulative coverage maps are provided by the public data catalogue^24^ of the Earth Observation Data Centre (EODC).

### Sentinel-1 mission ground coverage

As obvious from the density map in Fig. 1b,the mission’s coverage is inhomogeneous in two respects: First, ESA’s Sentinel-1 acquisition strategy prioritises Europe and tectonic active areas, whereas over other regions the CSAR data acquisition is selectively switched active/inactive, with the aim to make optimal use of the SAR cycle within the technical constraints of the overall system. Second, the effective ground coverage varies within a region, with either a rhomboid footprint pattern bound to the interwoven orbit overpasses in case of a dense coverage, or a linear pattern in case of coverage by only one local orbit overpass with CSAR data acquisition active.

Let us examine the overpass pattern in detail: It is persistent in time and stems from the fixed orbit- and observation-configuration with a repeat cycle of exactly 12-days for each Sentinel-1 satellite. The global data coverage was gradually improved during the mission’s exploitation ramp-up phase (covering our 2016–17 period), in line with the increasing operational capacity. Full details on the coverage pattern are outlined in the Sentinel-1 SAR observation scenario^25^. As a summary here, the scenario follows the Sentinel High Level Operations Plan (HLOP^26^) and defines for land monitoring that most of the global landmasses are mapped every 12 days at least. The European land and coastal waters are systematically mapped within the 6-day constellation repeat cycle in both ascending and descending passes, using dual-polarisation (VV & VH). Tectonic/volcanic areas outside Europe are covered alternating between ascending and descending passes, each within a 24-day repeat-pass interval. Specific observation campaigns and monitoring modes are set up for the global sea-ice and iceberg waters, and ice-sheets of Greenland and Antarctica.

Effectively, individual ground locations are covered with different revisits, ranging from 9 to 1 local observations within 12 days. This so-called coverage frequency is in general improving with latitude (e.g. over Europe), but shows also a longitudinal component, leading to areas of high observation frequency next to areas of low observation frequency (as discussed in detail by Bauer-Marschallinger *et al.*^9^). The density map in Fig. 1b clearly shows the linear patterns in non-prioritised regions covered by only one or two overpasses with CSAR data acquisition active, e.g., over great portions of Africa, Australia, Russia, Canada, and the eastern sections of the Americas. These sections were particularly challenging to normalise for the incidence angle. As a result, a remainder of the initial artefacts in the backscatter mosaics are visible in Fig. 1a over the tropic and subarctic forests.

### Mosaicking challenges

Overall, we faced two major challenges for the generation of harmonised global Sentinel-1 composite maps: First, aggregating a multitude of individual SAR scenes was imperative, as averaging over a large number of temporally distributed measurements was needed to suppress the impact of natural soil moisture variability and vegetation phenology. We had to collect and deal with a huge amount of Sentinel-1 input and interim data (a single Sentinel-1 scene has a compressed volume of about 1 GB), which needed to be preprocessed, structured, and stored in an efficient way. Second, as already mentioned above, it does not suffice to simply stitch the images together in time, since acquisition patterns from the differences in the viewing geometry persist. The next section is dedicated to describe how we tackled both tasks, presenting our chosen processing and data organisation strategy, and showing how one can normalise the Sentinel-1 data to reduce the influence of the observation geometry represented by the PLIA. The section closes with a description of our mosaicking procedure and quality curation.

### Data acquisition and preprocessing

Sentinel-1 Ground Range Detected High-resolution (GRDH) Interferometric Wide swath (IW) products are used as input for the S1GBM. The GRD data product type features a nominal spatial resolution of about 20 m × 23 m and contains dual-polarisation backscatter in VV and VH that has been detected, multi-looked, and projected onto the WGS84 ellipsoid with a 10 m ground sampling. IW is Sentinel-1’s main dataset over land that, however, leaves out large portions of Greenland, Novaya Zemlya, and the Canadian Arctic Archipelago (i.e. 2.1% of global land outside Antarctica). Those regions are observed by the Sentinel-1 mission in the Extra Wide (EW) swath mode at a 40 m projected sampling and in HH and HV polarisation (see the IW GRD data specifications^27^).

The Sentinel-1 IW data is collected and hosted by a dedicated service of the Earth Observation Data Centre for Water Resources Monitoring (EODC, https://www.eodc.eu/). To increase timeliness and completeness of the data collection, EODC deploys a so-called “Hubwatcher” to monitor and cross-check different data resources and hubs, e.g. the Copernicus Services Data Hub^28^ or the Sentinels Collaborative Data Hub^29^.

### Hard- and software setup

In the course of the development of the S1GBM, 460000 input Sentinel-1A/B scenes from the years 2016–17, totaling a raw data volume of 360 TB, were processed via 700 TB intermediate data to a total of 80 TB non-normalised and normalised mosaic layers. The Petabyte-scale data amount obviously placed high demands on processing- and storage- facilities, requiring hardware consisting of a supercomputer connected to a fast accessible mass data storage, and innovative processing chains to perform parallel processing of the manifold input sensor records and higher-levels intermediates.

These hardware requirements were met by the EODC/TUW’s infrastructure that employs 1) the Vienna Scientific Cluster 3 (VSC-3) embedding 2200 computing nodes each with 2 × 2.6GHz Intel processors and 64 GB or optionally 128 GB memory, 2) a VSC-side 0.6 PB BeeGFS Filesystem and EODC’s IBM GPFS storage holding 7 PB that is connected to VSC-3 via a 8 × 40 GBit InfiniBank fabric, and 3) an on-site virtualised research and development cloud environment. On this system, a total of 3 million core hours were consumed for the generation of the S1GBM.

Regarding processing software, the Department of Geodesy and Geoinformation at the TU Wien (Technische Universität Wien) developed the dedicated software suite SAR Geophysical Retrieval Toolbox (SGRT v2.4) for the large-scale processing of Sentinel-1 products and the subsequent data aggregation and extraction of geophysical parameters. Its framework uses the Python language and comprises workflows of several image and signal processing components, including a parallelised SAR preprocessing module environing the Sentinel-1 Toolbox (S1TBX^30^) of ESA’s Sentinel Application Platform (SNAP v6.0).

For storing and managing the processed data and final mosaics, we used the EODC/TUW’s global datacube approach based on the Equi7Grid^31^, a spatial reference system designed to handle efficiently the archiving, processing, and displaying of high resolution raster data over land. It preserves geometric accuracy and minimises data oversampling to a very low value of 2% (as compared to 36% of the Lat-Lon-projection, for example). Recently, the Equi7Grid was found to preserve the accuracy of geometric-analytical measures around the globe, being most beneficial for terrain analysis^32^. Defined for the entire Earth (code and data openly accessible on GitHub^33^), it consists of seven planar subgrids for each continent and features a scalable tiling scheme. In combination with an image stacking layout and a metadatabase at the storage file system, the Equi7Grid lays the foundation for a tiled datacube that directly enables parallelisation of processing and spatio-temporal data access required for the generation of the S1GBM aggregation layers.

### Sentinel-1 CSAR preprocessing

The preprocessing of Sentinel-1 SAR data constituted the by far most computation effort in the entire processing chain, and was the major driver to employ the described hard- and software system, with its large-scale computing cluster, efficient job parallelisation, fast file system, and capacious data storage.

SGRT’s preprocessing module ingests individually the Sentinel-1 IW GRDH images, and subsequently applies 1) orbital state vectors 2) image border noise removal following an algorithm developed specifically for S-1^34^ 3) annotated radiometric calibration factors 4) slant range – zero Doppler geometric terrain correction (using a combined 3arcsec SRTM/GDEM elevation model [VFP SRTM DEM at 90 m pixel sampling^35^]) 5) projection onto the Equi7Grid using gdalwarp^36^ with bilinear resampling and splitting into 100 km-sized “T1”-tiles.

The output images hold *σ*° (sigma nought) backscatter coefficient values in decibel (dB) and are time-stacked per Equi7grid-T1-tile, building the data basis for the mosaic generation. More details on the SAR preprocessing with SGRT can be found in dedicated studies^34,37,38^.

### Incidence angle normalisation

In general, the radar backscatter is the portion of a transmitted radar signal that is scattered back by observed objects. The received energy depends not only on the ground target itself and its characteristics comprising geometry, dielectric properties, and other (sub-)surface properties, but also on specifications of the sensor, such as radar frequency, polarisation, and the observation geometry. The Earth-sensor geometry is defined foremost by the local incidence angle (LIA), or more preferable the projected local incidence angle (PLIA), which accounts for the orientation of the local normal vector in azimuth and is herein referred to as *θ*^39^.

While sensor specifications are invariable and well-known, *θ* is variable and also substantially impacts the backscattered signal. Generally, backscatter decreases with increasing *θ*, since more energy is scattered forward than backward and is therefore not received by the sensor. SAR imaging sensors, with their side-looking observation geometry, intrinsically record the backscatter signal with a gradient from near- to far-range, which adds to a second geometric impact from the topography relief. While the effect from the relief could be accounted for through the reconstruction of the illuminated surface area following the approach of Small *et al.*^40^ for radiometrically terrain-flattened gamma nought backscatter (${\gamma }_{T}^{0}$), the backscatter’s dependency on *θ* due to different scattering mechanisms in different land cover types still needs to be modelled. Moreover, we aim for a global *σ*°-reference for future C-band missions. Therefore, to make all measurements from the Sentinel-1 imagery directly comparable—which is the main goal of mosaicking—we account for the different values of *θ* and normalise the *σ*°-backscatter recordings of each observation to a fixed reference angle.

Yet, modelling the relation of *θ* with backscatter expressed as *σ*° is not straightforward as it is a function of local properties of the observed surface. In case of a smooth surface, a specular reflection leads to a strong dependency. In case of a surface covered by vegetation, backscatter directionality is more uniform over all *θ*-angles due to volume scattering along the ray path, which leads to a weaker dependency of backscatter with *θ*. Seen from the opposite perspective, the PLIA-dependency carries useful information about land cover, vegetation, and changes within the phenological cycle^41^. Throughout the years, various *σ*°-backscatter normalisation techniques have been developed for SAR applications. Most of them are data-driven and estimate a regression model based on either a temporal dataset^42^ or a specific sensing geometry^43^. The more recent study in^9^ presents for 500 m-sampled Sentinel-1 IW data a multivariate linear regression approach that exploits local signal variation and mean backscatter. A more comprehensive approach^44^ takes into account also azimuthal modularisation, using the orientation of the local relief and orbit-specific statistics, and presents a new method to simultaneously normalise for the incidence- and the azimuth- angle of Sentinel-1 measurements.

The Sentinel-1 mission features—in support of repeat-pass SAR interferometry—a very stable orbit geometry repetition with well-defined orbital baselines, allowing the application of a-priori PLIA values *θ*_*ro*_, stored for each of the 175 relative orbits. In IW mode, Sentinel-1 covers incidence angles ranging from 29° to 46° using an Earth ellipsoid as reference surface. Aiming for a homogeneous global mosaic that is normalised to the central value of *θ* at 38°, we examined existing methods and concluded that a simple linear regression model is most robust, estimating per 10 m-pixel one single slope parameter for the local PLIA-dependency that describes the inverse linear relationship between *θ* and backscatter *σ*° (as in Pathe *et al*.^7^). This slope, or *β*, is then used in the normalisation step to tilt the local backscatter distribution (from the local time series *σ*°(*θ*, *t*)) to the reference incidence angle of 38°, yielding the normalised backscatter values *σ*° (38, *t*):

1 $${\sigma }^{0}(38,t)={\sigma }^{0}({\theta }_{ro},t)-\beta \,({\theta }_{ro}-3{8}^{\circ })\quad {\rm{[dB]}}$$

We note that an interpolation method based on historic Envisat ASAR Wide Swath data showed good performance over Europe, but could not be employed globally due to ASAR’s incomplete global coverage. Methods that estimate normalisation parameters for each Sentinel-1 orbit geometry showed much more computational effort while failing in areas observed from only one or two orbits, which have only little PLIA-spread. In those problematic areas, which are found globally but mainly in low-latitudes (compare with Fig. 1b), also our simple linear regression cannot produce reliable *β*-estimates. However, we found that a static value for *β* of −0.13 dB/° showed visually acceptable results in these areas and is used there in the S1GBM. This value is obtained as spatially averaged *β* from selected European areas that are covered by four Sentinel-1 orbits that span typically a PLIA-spread larger than 8°. While the use of a static *β*-value is not optimal, it was a practical choice that allowed us to complete the world-wide processing. In the future, this limitation might be overcome by improved Sentinel-1 coverage and machine learning approaches that predict spatially variable values for *β* in these areas using models trained in the well-covered regions.

### Layer aggregation & image mosaicking

Basically, the S1GBM mosaic layers are generated as statistical parameters from normalised backscatter derived from SAR data acquired in the period 2016–2017. After populating the global Sentinel-1 datacube with output from preprocessing the 460000 IW GRDH scenes, two sets of mosaics were generated, one from non-normalised backscatter for each relative orbit, and one from normalised backscatter combining all orbit overpasses. The latter builds the here presented mosaic layers and constitute the S1GBM Version 1.0. As a note, we also generated a separate set of mosaics for the Arctic region based on 40 m-sampled Sentinel-1 Extra Wide Swath (EW) mode data, but we omitted them in this first release for consistency reasons.

Within the 10m-sampled Sentinel-1 datacube, for each pixel the normalised backscatter time series *σ*° (38, *t*) were built, analysed, and processed to statistical parameters, such as mean, standard deviation, minimum, maximum, and counts of valid observations.

More specifically, the S1GBM mosaicking process was carried out individually per Equi7Grid T1-tile, consisting of the following steps:

1. selection and quality check of Sentinel-1 preprocessed data, comprising backscatter (i.e. *σ*°) and PLIA (i.e. *θ*) image stacks
2. generation of mean PLIA images per Sentinel-1 relative orbit, representing the a-priori *θ*_*ro*_ for 1–8 relative orbits covering a T1-tile
3. estimation of local slope (*β*) per pixel from linear regression, and filling up with *β* = −0.13 dB/° where the number of local relative orbits is ≤2
4. normalisation of *σ*° (*t*) to *σ*^0^ (38, *t*) with Eq. 1
5. conversion of *σ*^0^ (38, *t*) backscatter to linear unit
6. calculation of statistical parameters using the NumPy library^45^
7. conversion of statistical parameters to decibels
8. storing each parameter to a datacube, as a T1-tiled GeoTIFF file

### Artefacts and remedies

During the development of the mosaics, several issues and artefacts have been encountered with the Sentinel-1 IW GRDH inputs, such as 1) narrow, linear gaps between subsequent slices of Sentinel-1 data takes, 2) so-called “dirty pixels” on the border of an acquisition, 3) Radio-Frequency Interference (RFI) pollution, 4) bands of different backscatter levels due to thermal noise, and gaps at the antimeridian (i.e. 180° East/West).

The gaps between subsequent slices were initially found in the non-normalised parameters during the quality checking. Boosting efficiency and data downlink, ESA slices Sentinel-1 IW observations along-track per 25 sec sensing time. Those slices are published individually and share no overlap with their adjacent slices. However, during the geocoding step, computing the correct backscatter values along the cut-line-rows requires the adjacent measurements, or otherwise it generates no-data values, locally. As the SAR datasets are generally ingested separately and processed in parallel, narrow stripes of no-data are generated at a dataset’s start- and end rows, yielding linear gaps in the mosaic of one orbit data take, with a width up to 30 pixels. In most cases, this did not affect the integrity of the S1GBM’s mosaics, as the along-track position of the image slicing was not always the same, and hence the gaps were filled-up over time thanks to a sufficient input data density. In the case of the normalised mosaics, this is even more relaxed, as they combine data from up to six different orbits. However, areas covered by only one relative orbit were still impaired by those gaps, and this compelled us to implement a simple remedy: First we detected the artefacts (totaling 760 in non-normalised mosaics; 65 in normalised mosaics) using simple spatial operators. Second, we filled up the parameters with backscatter data by re-processing identified slices that we merged pair-wise before geocoding.

One challenging problem was caused by SAR measurement artefacts, which required dedicated manual quality curation of our team. Effects such as Radio Frequency Interference (RFI), and “dirty pixels” stemming from erroneous resampling in the GRD data, occur both randomly in the IW GRDH data and are difficult to detect with an automatised procedure. To screen these artefacts, downsampled quick-look images at various scales have been generated and checked manually. The clean and final S1GBM parameters were generated then by excluding artefact-carrying backscatter images.

Thermal noise is an additive background noise that is caused by microscopic motion of electrons due to SAR instrument temperature and can be observed when backscatter is very low (<−22 dB), e.g. over water or deserts and at VH-polarisation. Sentinel-1 IW (and EW) measurements are multi-swath observations and suffer from unequal noise effects in the sub-swaths, leading to discontinuous sharp changes in the intensity at inter-swath boundaries^46^. The different noise levels in the IW data-takes can by corrected through application of annotated thermal noise vectors, but only with the Sentinel-1 Instrument Processing Facility (IPF^47^) upgraded to version 2.90 in March 2018 thermal noise could be reduced significantly, and has been much less apparent since then. Consequently, in our 2016–17-based S1GBM, linear artefacts from thermal noise might be observed over areas with low backscatter, in particular over low-backscatter-areas like waters or deserts in the VH-mosaic.

Another problem occurred during the preprocessing of datasets that cross the antimeridian. These data sets could not be pre-processed successfully with SNAP v6.0 in the default Lat-Lon projection (to which SNAP’s geocoding module is optimised), simply due to discontinuity at the antimeridian. Therefore, the Azimuthal Equidistant projections for Asia and North America of the Equi7Grid system were directly used during the preprocessing of the affected datasets.

## Data Records

The Sentinel-1 Global Backscatter Model (S1GBM) describes the C-band radar cross section of the Earth’s land surface of the years 2016–17, generated from Sentinel-1 IW GRDH backscatter normalised to the reference incidence angle of 38°. In the Version 1.0 presented here, the S1GBM comprises two global mosaics describing the temporal mean of sigma nought backscatter *σ*^0^ (38, *t*) in VV- and VH-polarisation respectively.

### Dataset structure

Analogous to the Sentinel-1 preprocessed input datacube, each mosaic is sampled at 10 m pixel spacing, georeferenced to the Equi7Grid^31^, and divided into six continental zones (Africa, Asia, Europe, North America, Oceania, South America, omitting Antarctica), which are further divided into square tiles of 100 km extent (“T1”-tiles). With this setup, the S1GBM consists of 16071 tiles over six continents, for VV and VH each, totaling to a data volume of 2.67 TB. For the purpose of this publication, the dataset is organised in twelve collections, one for each of the six continents and the two polarisations. See Table 1 for the summary of the collections’ file names, tile counts, and data volume.

**Table 1:** The ESA S1GBM data publication is organised at the TU Data repository in twelve collections, with information on tile count per continent and polarisation, and data volume.

Collection file name	Continent	Tiles VH	Tiles VV	Volume
S1GBM_VH_mean_mosaic_v1_EQUI7_AF010M	Africa	3775		320 GB
S1GBM_VV_mean_mosaic_v1_EQUI7_AF010M			3776	336 GB
S1GBM_VH_mean_mosaic_v1_EQUI7_AS010M	Asia	4457		378 GB
S1GBM_VV_mean_mosaic_v1_EQUI7_AS010M			4457	379 GB
S1GBM_VH_mean_mosaic_v1_EQUI7_EU010M	Europe	1339		100 GB
S1GBM_VV_mean_mosaic_v1_EQUI7_EU010M			1339	98 GB
S1GBM_VH_mean_mosaic_v1_EQUI7_NA010M	North America	2669		223 GB
S1GBM_VV_mean_mosaic_v1_EQUI7_NA010M			2670	215 GB
S1GBM_VH_mean_mosaic_v1_EQUI7_OC010M	Oceania	1786		144 GB
S1GBM_VV_mean_mosaic_v1_EQUI7_OC010M			1788	139 GB
S1GBM_VH_mean_mosaic_v1_EQUI7_SA010M	South America	2041		169 GB
S1GBM_VV_mean_mosaic_v1_EQUI7_SA010M			2041	169 GB
	Total	16067	16071	2.67 TB

Note that for each of the 32138 tiles, an additional quicklook-file is provided, yielding a total number of 64276 files.

### Dataset publication

The dataset is published by ESA and hosted by the TU Wien Data Repository platform (TU Data), an institutional data repository for publication of data following the FAIR principles^48^. The S1GBM dataset and its specifications can be found on

- on its ESA landing page^49^
- and via its Digital Object Identifier (DOI)^50^

All of the twelve collections share this single DOI, but can be downloaded individually as zipped folders (.zip). The long term availability of data is one of the core objectives of the TU Data repository, and the S1GBM data record will be maintained for a minimum of 10 years.

The tiles’ file-format is a LZW-compressed GeoTIFF holding 16-bit integer values, with tagged metadata on encoding and georeference. Compatibility with common geographic information systems as QGIS or ArcGIS, and geodata libraries as GDAL is given. In addition to the original data images, downsampled quicklook-files of same geographic extent are available for all tiles, allowing previewing the data in a convenient manner.

### File nomenclature

The folder logic within a collection may be illustrated by the example of North America, tile “E064N036T1” and VH polarisation:


\S1GBM_VH_mean_mosaic_v1_EQUI7_NA010M\E064N036T1\


A filename of one tile of a mosaic may be for example:


M20160104_20171230_TMENSIG38_S1-IWGRDH1VH-_——_B0104_NA010M_E064N036T1.tif


It defines the following:

- “M” for the actual main data, or “Q” for the quicklook-file (for preview, see below).
- start- and end-time of input data to this mosaic tile, in the format YYYYMMDD
- the aggregated statistical parameter; for Version 1.0 this is always “TMENSIG38”, i.e. mean of backscatter normalised to 38°
- relating to the input data, the satellite and sensor mode identifier “S1-IWGRDH1”, abbreviating Sentinel-1 Interferometric Wide Swath mode that is Ground Range Detected at High-resolution
- the backscatter polarisation; so “VV” or “VH”
- the version of TU Wien’s internal processing engine, i.e. “B0104”
- the identifier for Equi7Grid’s continental grid, with pixel sampling in meters, e.g., “NA010M” for North America and 10 m pixel size
- the identifier for Equi7Grid’s tile within the continent, defined by the lower left coordinate, and the tile extent; e.g. “E064N036” for 6400 km easting and 3600 km northing, and “T1” for 100 km tile extent to the east and north

### Web-based data viewer

In addition to the data provision at the TU Data repository^50^, we set up at the EODC facilities an open web-based dataset viewer^51^, offering an intuitive pan-and-zoom exploration of the full S1GBM VV and VH mosaics. It was designed for use by both laymen and professional users who want to quickly browse the S1GBM, allowing them to get a visual impression of the mosaics. To ease browsing the dataset accessed from the repository, the EODC/TUW data viewer features a graphical overlay of the continental zones and the T1-tiles of the Equi7Grid.

## Technical Validation

With S1GBM’s characteristics as a global, PLIA-normalised, high-resolution C-band backscatter dataset, a direct validation experiment is not feasible since we lack matching reference backscatter data collected during airborne or ground based radar campaigns. Other existing global mosaics were generated based on different time-spans, polarisations^17^, frequencies^18^, or do not share the novel feature of the PLIA-normalisation^20^.

On these grounds, we prefer to assess the characteristics of the S1GBM layers with respect to different land cover types on a global scale, and to incorporate the gained knowledge into an easy-to-use classification algorithm for permanent water bodies (PWB). This simple mapping experiment acts as an example and should on the one hand demonstrate the integrity and quality of the S1GBM mosaics (and document its limitations), and on the other hand, stimulate more advanced applications and ingestion-models by the remote sensing- and the wider user -communities. Our validation of the obtained PWB-map compares—over a representative and diverse set of eight world regions (see Fig. 1b)—the S1GBM mosaic with a reference water body map, as well as with true-colour imagery from the Sentinel-2 optical sensor. This arrangement should also portray the shape and texture of the S1GBM mosaic and help the audience with the interpretation of the SAR imagery, which as stated at the outset, allows a unique view on the Earth’s surface.

In the following, 1) we examine in detail the appearance and spatial features of the S1GBM VV- and VH-mosaics over the region of Bordeaux, also investigating the effect of the PLIA-normalisation. Then, 2) we derive the characteristic C-band backscatter signature for global land classes. Finally, 3) we perform the PWB-experiment in eight world regions a) to evaluate the dataset’s integrity, b) to demonstrate its spatial information and exemplify its use, and c) to comment on the S1GBM’s assets and caveats.

### Detail example Bordeaux

Figure 2 gives an example of the land cover signal in the S1GBM VH and VV mosaics over Bordeaux, France. Comparing it with the recent PROBA-V-based Land Cover dataset of the Copernicus Global Land Service (CGLS LC100^52^), several surface features are apparent in the mosaics, including urban areas with varying density in both VV- and VH-channels. In the VH mosaic, a clear discrimination of forest areas (cf. with LC100’s broadleaf in brighter green, needle leaf in darker green) against crops (brighter yellow) and vineyards (darker yellow) is apparent. The cross-polarised VH-backscatter is more sensitive to vegetation-density, -structure, and -status, as multiple scattering between branches and volume scattering increases the share of backscattered microwaves with changed polarisation. Most prominent, in both VH and VV, is the very large contrast between land surfaces and open waters with significant lower backscatter signatures. This is the basis for our PWB-mapping experiment discussed in detail in the subsequent section.

We would also like to draw the attention to the spatial detail carried by the S1GBM mosaics, with various features at deca- and hectometric scale shown for example in Fig. 2. For instance, one can see bridges, highways, railways, and airports in the Bordeaux metropolitan area in the south-west corner of the here displayed T1-tile (100 km extent). Also, in the west, from north to south, the shorelines of the Gironde estuary and its downstream rivers are clearly mapped, resolving small islands and narrow straits. Agricultural plots and forest sections may be differentiated especially in the VH mosaic, e.g. with particular structures in the north-west corner. For further exploration, users may visit the open web-based S1GBM viewer^51^ offering a pan-and-zoom exploration of the full S1GBM VV- and VH-mosaics.

Figure 2b,d allows the comparison of the S1GBM VV backscatter mosaic (which underwent the PLIA-normalisation) against the mean of non-normalised Sentinel-1 VV backscatter from the same observation period (not part of the dataset publication; just for comparison). As discussed above, radar backscatter is strongly dependent to PLIA, and hence Sentinel-1 SAR images are subject to the observation geometry defined by the mission’s relative orbit configuration and the overlapping pattern (cf. global map in Fig. 1b). One can clearly see this impact in Fig. 2d, where data from all local orbits are averaged in their native orbit geometry (i.e. mean of *σ*^0^ (*θ*_*ro*_, *t*), resulting to characteristic linear artefacts of backscatter discontinuities along the limits of the (repeating) orbit footprints. The mini-map of the Bordeaux-T1-tile in Fig. 2d plots the number of input Sentinel-1 scenes, also reflecting the heterogeneous coverage pattern induced by the different number of overlapping relative orbits (from 2 to 4 in this area), each with a different local PLIA-range, generally. Notably, the triangular zone covered by only 2 orbits (yellow, 194 scenes) is a zone that features a PLIA-spread that is not large enough to reliably estimate the local PLIA-slope *β*. This zone is part of the pixel domain where we applied the static slope value of −0.13 dB/° to the S1GBM mosaic, with a resulting backscatter image that is free from orbit-related artefacts (Fig. 2b). We note that the sections covered by 3 or 4 orbits in this example are normalised with the regular regression slope, letting us conclude that our approach yields a smooth mosaicking impression in areas of mixed coverage density.

### Backscatter signature analysis

Delving into above concept that SAR backscatter characteristics in the S1GBM are determined by land cover, we analysed the backscatter signature for the global land surface for each major land cover class (LCC). We globally aggregated data from the normalised S1GBM VV and VH mosaics per LCC and formed the backscatter distribution within each LCC, allowing the discrimination of typical SAR backscatter signatures for a specific land cover class.

#### Land cover definitions

As land cover dataset, we selected the above-mentioned PROBA-V-based CGLS LC100 for its full global coverage and the (for global datasets) relatively high spatial resolution with a pixel spacing of 100 m. To allow a fast pixel-by-pixel comparison, we resampled the CGLS LC100 to the Equi7Grid at 10 m using nearest-neighbour-downsampling. After a first inspection of backscatter signatures, we grouped the 23 LCC of the LC100 to 13 major LCC, accounting for the similarity between certain classes: Respective *open* and *closed* forest classes were aggregated to *evergreen needle leaf forest*, *evergreen broad leaf forest*, *deciduous needle leaf forest*, and *deciduous broad leaf forest*, and *herbaceous wetland* was grouped with *herbaceous vegetation*. Table 2 lists the main statistics per land cover and the group aggregations.

**Table 2:** Sentinel-1 backscatter statistics per land cover class (LCC) of the CGLS LC100 dataset, mean and standard deviation, for the S1GBM mosaics in VV and VH polarisation.

ID	Name	Mean ${{\boldsymbol{\sigma }}}_{{\boldsymbol{V}}{\boldsymbol{V}}}^{{\boldsymbol{0}}}$	Mean ${{\boldsymbol{\sigma }}}_{{\boldsymbol{V}}{\boldsymbol{H}}}^{{\boldsymbol{0}}}$	Std ${{\boldsymbol{\sigma }}}_{{\boldsymbol{V}}{\boldsymbol{V}}}^{{\boldsymbol{0}}}$	Std ${{\boldsymbol{\sigma }}}_{{\boldsymbol{V}}{\boldsymbol{H}}}^{{\boldsymbol{0}}}$
0	No input data available	−10.54	−18.71	4.38	5.75
111	Closed forest, evergreen needle leaf	−10.20	−16.16	1.58	1.71
113	Closed forest, deciduous needle leaf	−11.61	−18.18	1.30	1.53
112	Closed forest, evergreen, broad leaf	−8.08	−14.00	1.66	1.47
114	Closed forest, deciduous broad leaf	−9.63	−15.35	1.57	1.61
115	Closed forest, mixed	−9.84	−15.69	1.18	1.24
116	Closed forest, unknown	−10.22	−16.45	2.37	2.73
121	Open forest, evergreen needle leaf	−11.00	−17.23	1.97	2.16
123	Open forest, deciduous needle leaf	−12.30	−19.13	1.69	2.06
122	Open forest, evergreen broad leaf	−9.21	−14.93	2.11	1.90
124	Open forest, deciduous broad leaf	−10.52	−16.31	1.86	1.87
125	Open forest, mixed	−10.32	−16.25	1.64	1.73
126	Open forest, unknown	−10.85	−16.88	2.23	2.49
20	Shrubs	−12.32	−18.52	2.60	2.93
90	Herbaceous wetland	−13.28	−20.86	2.76	3.36
100	Moss and lichen	−11.89	−20.87	2.96	3.65
40	Agriculture	−11.87	−19.03	2.21	2.81
50	Urban/built up	−7.94	−15.02	3.28	2.89
70	Snow and ice	−9.16	−15.73	5.07	6.05
30	Herbaceous vegetation	−13.71	−21.05	3.06	3.63
60	Bare vegetation	−15.75	−22.98	5.17	4.36
200	Open sea	−18.85	−28.28	2.15	1.75
80	Perm. water bodies	−18.85	−26.42	2.53	2.28
—	Evergreen needle leaf forest	−10.25	−16.23	1.62	1.76
—	Deciduous needle leaf forest	−11.61	−18.19	1.30	1.54
—	Evergreen, broad leaf forest	−8.09	−14.01	1.67	1.48
—	Deciduous broad leaf forest	−9.85	−15.61	1.63	1.67
—	Herbaceous vegetation	−13.68	−21.04	3.04	3.62

Classes combined in this study are shown without a LC100 ID value.

#### C-band backscatter signatures

**Fig. 3:**
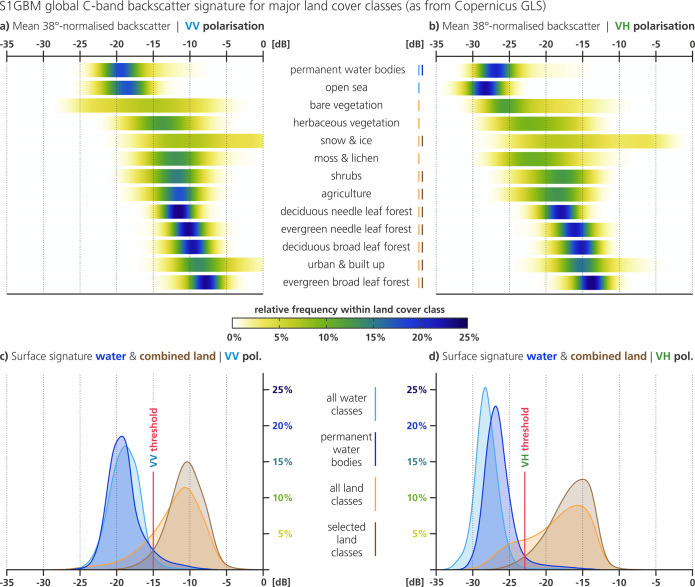
Results from the S1GBM C-band backscatter signature analysis for major land cover classes, which are provided by the 100 m Land Cover Version 2.0 product of CGLS. The heatlines in (**a**) and (**b**) show the S1GBM’s normalised backscatter distribution within the total area of each major land cover class, for VV and VH, respectively. In preparation for the mapping of permanent water bodies (PWB), (**c**) and (**d**) show the distributions for the globally combined water- and land- surfaces, with the combined classes indicated by blue and brown bars in (**a**) and (**b**) legends. For the PWB-mapping, three land cover classes have been excluded due to the lack of clear separability against the water classes, i.e. due to largely overlapping distributions. The selected thresholds for VV and VH mosaics used in our PWB-mapping algorithm are indicated as red lines.

The C-band backscatter signatures of our major 13 LCC are plotted for VV- and VH-polarisation as distribution-density-“heatlines” in the upper part of Fig. 3, illustrating the global average backscatter levels of each surface class, and the variance within. Forest and water-body classes have a very narrow distribution, whereas *snow and ice* and *bare vegetation* have a greater spatial backscatter variability. Snow and ice packs often have a heterogeneous structure from its complex genesis involving melting and freezing phases, leading to a mixture of surface- and volume-scattering when observed by radar. Likewise, the LCC *bare vegetation* comprises very different surfaces dominated by rocky, sandy, or mountainous surfaces, each governed by a distinct backscatter behaviour and hence create the wide spread within this LCC.

The LCC-heatlines in Fig. 3a,b are approximately ordered by the mean backscatter value. On top, one can find the two water LCCs with a very low backscatter level that is caused by mirror-like-reflection away from the sensor, followed by bare and herbaceous vegetation LCCs that are dominated by dry conditions and hence are generally weak scatterer. The LCCs *moss & lichen*, *shrubs*, and *agriculture* feature medium backscatter and variation thereof. Higher backscatter levels are observed over the *forest* LCCs, where volume and multiple scattering become more dominant, as well as over the LCC *urban & built up*, where corner reflections acting as echo cause the strongest radar backscatter.

When comparing VV and VH polarisation, the biggest difference is in the overall level of backscatter, with about 7 dB between both polarisations across all LCCs. The order of LCCs as a function of mean backscatter is mostly the same for VV and VH, except for the water and ice classes. Interestingly, the *open sea* class shows a steeper drop from VV to VH, whereas *shrubs* show a comparatively small drop. We found that the strongest changes in the backscatter distributions are apparent in the non-forest vegetation classes, e.g. for *bare vegetation* and *agriculture*, supporting our initial assumptions on the sensitivity of Sentinel-1 VH backscatter to complex vegetation dynamics and crop varieties.

### Permanent water body mapping

Following up to what we have already seen along the rivers in Fig. 2, water bodies (represented by the LCCs *open sea* and *permanent water bodies*) show a most distinctive backscatter signature in relation to other land cover classes (cf. 3a-b). Effectively, water surfaces show in radar images a strong contrast with land surfaces. The reason for this are the different microwave scattering mechanism over water- and land-surfaces and the side-looking geometry of SAR systems. A specular reflection of the radar pulses by the water surfaces leads to backscatter intensities received by the sensor that are much lower than for most other land cover types. With the S1GBM VV- and VH-mosaics at hand, we exploited this discriminative feature of water bodies and employed a simple permanent water body mapping method. Unlike the backscatter mosaics of the S1GBM, the obtained PWB map can be validated directly, as we have available matching global water body maps as a reference. Moreover, the experiment should demonstrate the ease of realising a land cover mapping application in short time, exploiting the novel S1GBM data and its high-resolution radar imagery of the Earth’s land surfaces.

Based on above insights from the Sentinel-1 backscatter signature analysis, our first step was to spatially merge all water- and all land-LCCs and build the combined backscatter signatures for VV and VH (Fig. 3c,d). The water distribution (*all water classes*; bright blue) is plotted for both polarisations next to the non-water distribution (*all land classes*, bright brown), already demonstrating an acceptable feature separation. However, as one can see in the heatlines above, water has still some significant overlap with some land LCCs, e.g. with *bare vegetation*, *herbaceous vegetation*, and *moss & lichen*. Naturally, this translates to a considerable overlap in the merged distributions below, especially in the VH case and for *moss & lichen*. We concluded that for these LCCs no robust separability against water bodies is given in the S1GBM data and excluded the three classes from further PWB-mapping. Also, we dropped the LCC *open sea* in further processing as we limit the PWB experiment to inland surfaces (that are also covered by the reference dataset). The backscatter distributions of the PWB LCC and the selected land LCCs are shown in dark blue and brown (*permanent water bodies* and *selected land classes* in Fig. 3c,d), with a noticeably improved separability, especially in VH polarisation.

As a next step, evoking the theory of Bayesian inference with equal priors for binary classification, we obtained a statistically optimal global threshold for VV and VH, each. In this respect, we identified two thresholds, −15.0 dB for VV and −22.9 dB for VH polarisation, which we applied in a third step as an upper-bound backscatter-value on the complete S1GBM mosaics to map the global PWBs. Note again that the LCCs *bare vegetation*, *herbaceous vegetation*, *moss & lichen*, and *open sea* are not included in the PWB-mapping and are masked in all later results.

Although the VV and VH mosaics are redundant to some degree, the consideration of both channels is most advantageous for the PWB-mapping. First, the classification based on Bayesian inference is more robust when resulting from two discriminations. Second, while the VH mosaic offers a better separability between water and non-water (having less overlap in the distributions and hence less false positive and negative classifications), and the heatline of the PWB-LCC is better defined in VH, the VV mosaic offers in general a higher spatial detail due to its stronger backscatter signal and hence more favourable signal-to-noise ratio.

By applying the obtained thresholds to the normalised S1GBM mosaics as simple classification rules

2 $${\sigma }_{0}^{{\rm{VV}}}(38)\le -15.0\;{\rm{dB}}$$

3 $${\sigma }_{0}^{{\rm{VH}}}(38)\le -22.9\,{\rm{dB}}$$

and through joining them with logical “AND”, we were able to produce a global PWB map in less than two hours, using 70 parallel cores on the VSC-3 supercomputer.

### Evaluation of S1GBM mosaics and PWB map

To evaluate our S1GBM permanent water body (PWB) map, we chose as a reference dataset the Global Surface Water (GWS^23^) from the European Commission’s Joint Research Centre (JRC-EC). The GSW offers globally at a 30 m native sampling different variables on water bodies, e.g. annual seasonality, occurrence, recurrence, or maximum extent, and is based on 36 years of Landsat data in its newest version (GSW1_2). Although the annual seasonality for 2015 or 2016 was not accessible from version GSW1_2 at the time of writing this manuscript, we found the *Seasonality 2015* dataset of the GSW1_0 version suitable as a reference. Pixels valued with seasonality “12” (i.e. all months) are labelled *permanent water* and constitute our reference PWB map, which we warped by means of bilinear resampling to the Equi7Grid at a 10 m pixel spacing.

The evaluation presented in this paper was carried out on a representative and diverse set of eight world regions (see locations in Fig. 1b). For each region, classification results were assessed by a pixel-by-pixel comparison between the PWB map from S1GBM and from the GSW reference. Having such binary maps (water vs. non-water) it was straightforward to generate an “accuracy layer” representing the four elements of the commonly used confusion matrix, i.e. true positives, false positives, false negatives, and true negatives, to discuss the skill of the S1GBM to map PWBs. Areas belonging to the four excluded LCCs were masked in the result plots. Furthermore, to give some visual guidance in the evaluation regions, we acquired from the Copernicus Sentinel-2 Global Mosaic (S2GM) service the RGB-composite for the year 2019^53^ (the mosaic for 2015 was available only over Europe).

**Fig. 4:**
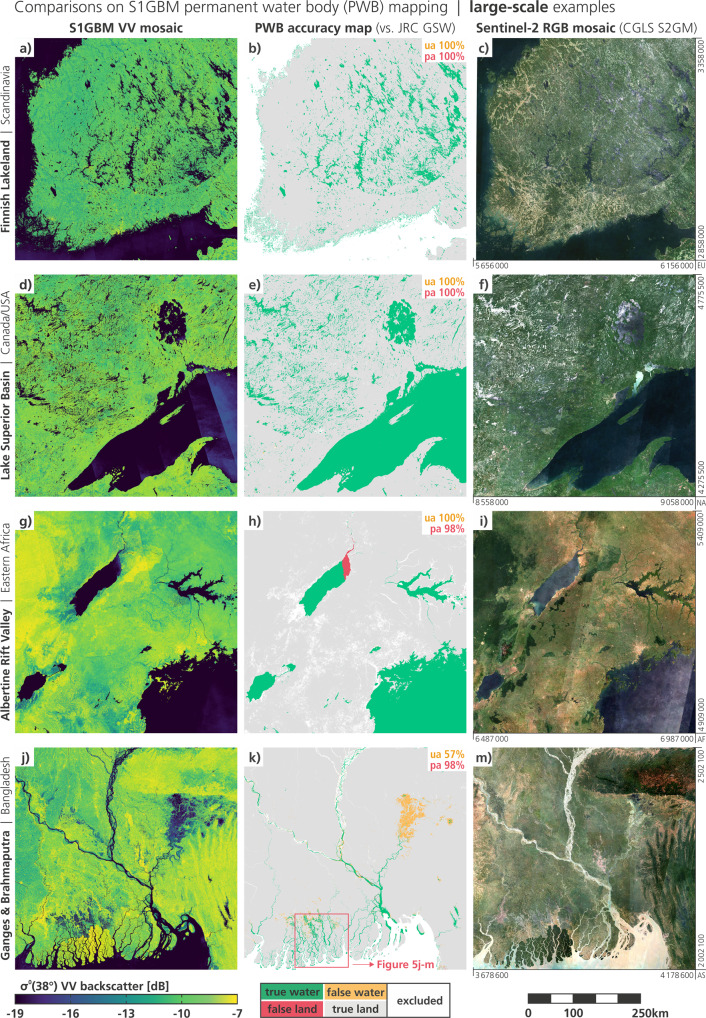
For four example sites at the large scale (500 km extent), the S1GBM VV mosaic (left) is contrasted with classification results from the S1GBM PWB mapping against the PWB taken from JRC Global Surface Water (GSW) in 2015 (centre), and with the RGB-composite of the Copernicus Sentinel-2 Global Mosaic (S2GM) for the year 2019 (right). Box outlines are shown in global overview in Fig. 1b.

**Fig. 5:**
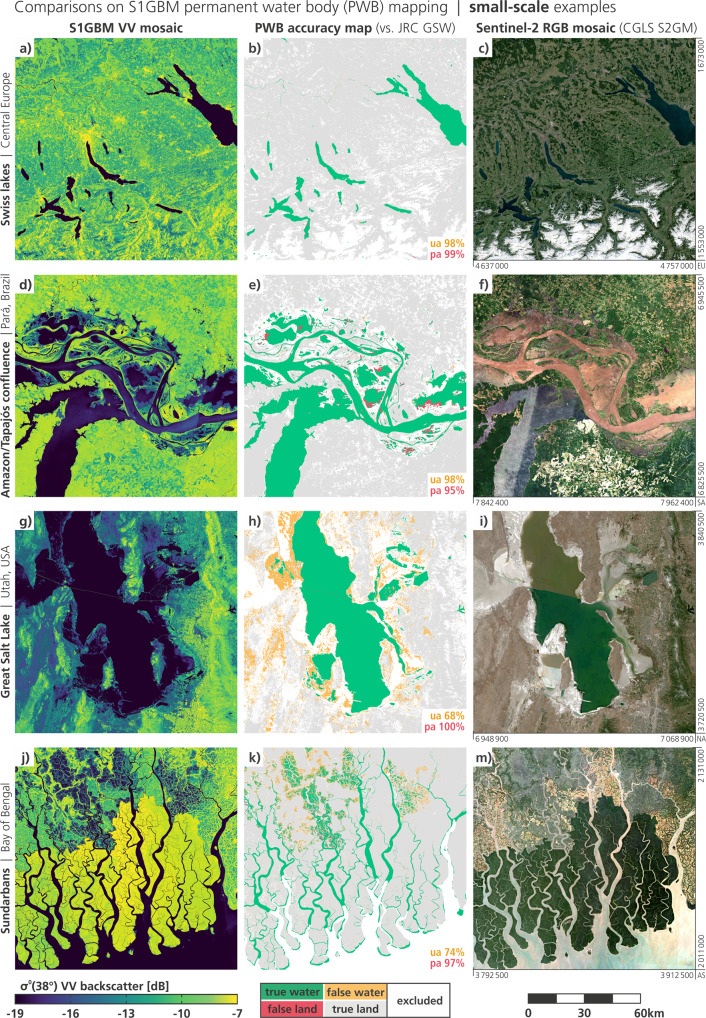
For four detailed example sites (120 km extent), the S1GBM VV mosaic (left) is contrasted with classification results from the S1GBM PWB mapping against the PWB taken from JRC GSW in 2015 (centre), and with the RGB-composite of the Copernicus S2GM for the year 2019 (right). Box outlines are shown in global overview in Fig. 1b.

In the following, we present results for four large-scale regions (500 km × 500 km) in Fig. 4, and for four small-scale regions (120 km × 120 km) in Fig. 5. For each region, the S1GBM VV mosaic is displayed on the left panel (space-saving/omitting the VH mosaic, which contributes likewise to the PWB mapping), the accuracy maps showing the performance against the GSW reference in the centre panel, and the Sentinel-2 RGB-composite to aid visual interpretation on the right panel. The accuracy maps are annotated with the respective User’s Accuracy (UA) and Producer’s Accuracy (PA), as the percentage of the agreement between the two PWB-maps.

#### Large-scale examinations

Figure 4a–c shows the southern part of Finland, an area accommodating a multitude of small and large post-glacial lakes. Those are clearly visible in dark colours representing low backscatter values in the S1GBM mosaic, while the other parts of the country (which is dominated by vast forests) shows rather uniform medium backscatter. The optical RGB-composite from Sentinel-2 does not feature the same accentuation of the lakes, troubled by remainders of cloud coverage in the yearly mosaic. The PWB accuracy map shows perfect agreement between S1GBM and GSW, with an UA and PA of 100% each. We identified two reasons for the excellent performance: First, the C-band backscatter signatures of the predominant land covers in Finland, such as forests, cities, agriculture, are well distinguishable against water bodies and hence allow an almost sterile PWB-mapping. Second, northern Europe is well covered by the Sentinel-1 mission and the S1GBM has been built with a high data density, letting us expect the best mosaic quality.

Moving to the region of the Lake Superior Basin in Canada and USA presented in Fig. 4d–f, we encounter a very similar, cold-temperate environment, but with a substantial higher share in spacious inland water bodies. Also here, the accuracy map shows a perfect agreement between S1GBM and GSW, which, in our interpretation, is clearly because of good feature separability in the SAR image. Particularly remarkable is that North America is much less covered by Sentinel-1 than Europe and that the imperfect modelling of the PLIA-dependency over water surfaces (as apparent e.g. in the east section of Lake Superior) does not impair the S1GBM PWB-mapping. Generally, imperfect PLIA-normalisation of SAR images is prominent over water bodies, whose specular reflection regime is characterised by a very strong PLIA-gradient (i.e. the slope *β*). However, we note that also the Sentinel-2 mosaic has striping artefacts bound to orbit footprints, and additionally suffers from cloud cover. The latter is a common problem in optical observation of higher latitudes, but is without effect in SAR imagery.

Figure 4g–i depicts the situation for a section of the Albertine Rift Valley in eastern Africa with its lake system. Reflecting to a great deal the region’s diverse flora, which is displayed in many green and brown tones in the RGB-composite, the S1GBM VV mosaic shows a much more heterogeneous pattern than in the above examples. The forested sections in the west show distinct higher backscatter values than the savanna sections in the east, and also other geomorphological features correspond well with the radar and optical mosaic. Concerning the PWB-mapping, we see again perfect agreement, but with one large exception: the eastern end of Lake Albert is entirely labelled in red as false land, suggesting that these water areas are missed in the S1GBM PWB map (what can be confirmed after a quick check with common thematic maps). In this area we see the impact of the relatively poor input data density of about only 50 Sentinel-1 scenes (cf. Figure 1b), and apparently, we overlooked the impact of a few images with outlying backscatter levels during the manual quality curation. Moreover, the three Sentinel-1 relative orbits covering this area create almost identical viewing angles and yield a very small PLIA-range, troubling our backscatter normalisation. As a result, striping artefacts appear not only over water bodies (cf. Canada example) but also over land (in north-west part Fig. 4g), while, however, the Sentinel-2 mosaic is likewise affected by striping issues (cf. Figure 4i), for other reasons, though.

The last row in Fig. 4(j–m) is centred at Bangladesh and displays the confluence of the Ganges and Brahmaputra streams, which are joined downstream by the Meghna river and ultimately discharge into the Bay of Bengal. Also in this region, the geomorphological features perceivable in the RGB-composite are reflected well by strong textural patterns in the S1GBM mosaic, promoting its broader use in land cover applications (note also the zoom-in plotted in Fig. 5j–m). The PWB-mapping results are inconclusive, as rivers of all sizes are correctly mapped, but many pixels are labelled in yellow as false waters. We consider this disagreement between S1GBM and GSW to be most likely a result of the different temporal resolutions of the two datasets, as the S1GBM is a two-year data aggregation reduced to single layers, whereas the GSW allows monthly snapshots of water bodies. For example, the Hoar ecosystem—which appears as yellow bulb in the north-east of Fig. 4k—is a large monsoon-fed lagoon system that is labelled by the GSW with seasonality-values ranging from 9 to 12 months. In the S1GBM mosaics, which are built using temporally averaged backscatter, these areas are obviously dominated by the high occurrence of water surfaces and act therefore as “most-of-the-time water bodies”. Some more vindication comes from the Sentinel-2 yearly mosaic, which also draws the Hoar area with a water texture. We conclude on this matter that seasonal water bodies are not properly modelled by our simple approach with Eq. 3, and it would need additional inputs from variance measures like the backscatter standard deviation.

#### Small-scale examinations

Figure 5 depicts the small-scale example regions with respect to the PWB-mapping experiment. The first row in a-c) zooms to the Swiss lakes in central Europe and both, the radar and the optical mosaic, feature a high level of heterogeneity and detail, with many individual forests, cities, valleys, rivers, alpine lakes, and with the airport north of Zurich resolvable (in the centre-left of the box). The results from the PWB-mapping are very good with high UA- and PA-values, but with two anomalies: First, the southern arm of Lake Lucerne (in the south-west) shows some red segments of false land along the mountain flanks reaching into the lake. After inspection of the S1GBM mosaics we can state that this is clearly an artefact from the terrain modelling with the rather coarse, 90 m-sampled VFP SRTM Digital Elevation Model (DEM) during the Sentinel-1 preprocessing. At the time of the project, we selected the VFP DEM^35^ for its complete global coverage and its manually-checked quality, and accepted the coarse resolution (with respect to the 10 m-sampled Sentinel-1 SAR data). The second small anomaly can be found in the Alps in the south of the image, with the west-end of the Klöntalersee labelled in yellow as false water. The S1GBM is artefact-free at this location, and after checking the GSW’s seasonality, we hypothesise that ice covers this mountain lake during winters and leads to the different interpretation.

Figure 5d–f presents the area around the confluence of the Amazon and Tapajós streams in central Pará in Brazil. Here, the rivers ramify into a multitude of lagoons and channels at various sizes, forming a complex system of water bodies. Fortunately, while the Sentinel-2’s RGB mosaic appears impure and rugged from contamination with the frequent cloud coverage in the central tropics, the Sentinel-1 mosaic offers a clear image that fully resolves the capillary structure of the water bodies and its shorelines. We consider this a remarkable feature, also recognising the very low input data density of the S1GBM mosaics in this area (cf. Figure 1b). Concerning the PWB-mapping, we obtained a good agreement with the GSW’s reference, labelling most PWBs correctly and misclassifying only small sections of the lagoons and river-arms. The false-water deviations are bound again to the seasonality of those segments that are most of the time under water, much alike to the situation in Bangladesh discussed around Fig. 4j–m. The red-labelled areas highlight water bodies which are mapped by the GSW but not by the S1GBM, and are of particular interest, as they exemplify that water surfaces seen by optical sensors are not necessarily identical to those seen by radars^54^. Swamp-like structures and waters with out-growing vegetation show a completely different SAR signature and hence might be distinguishable from open waters within a SAR image.

The third small-scale example is the Great Salt Lake in Utah, USA, as displayed in Fig. 5g–i. The S1GBM offers many details of Salt Lake City’s structures in the south-east, and of the mining facilities at the eastern shorelines of the lake, as also visible in the RGB-composite. Obviously, the radar image does not account for the difference in salinity between the north- and south-section of the Great Salt Lake that is visible in the optical image. However, our S1GBM PWB method maps correctly—contrary to the GSW reference—the east-west rail causeway splitting the lake, which one can see as a red line in the accuracy map in Fig. 5h. With its pronounced semi-arid climate, this region shows a different behaviour than above examples. The dry conditions and the sparse vegetation with its weak scattering trouble seriously the S1GBM PWB-mapping, with many false water pixel all around the area. Here, we see the weak performance of the simple threshold approach with Eq. 3 in regions with a general low backscatter from land, and hence small contrast to water bodies.

Figure 5j–m zooms into the Sundarbans at the southern shorelines of Bangladesh, with its multifaceted surface and its complex river-deltas. Both, the true-colour image from Sentinel-2 and the VV-mosaic from Sentinel-1 produce a feature-rich image and highlight the mangrove forest in the southern section with strong green colour or high backscatter, respectively. Adjacent to the north, the rice and bean agriculture draws large contrast patterns in the satellite images. For the PWB-mapping, a similar result as from the larger view on this region (cf. Figure 4k) is obtained, with all rivers and channels correctly classified, but with a substantial overestimation of permanent water bodies in areas of high water seasonality. To what extent rice fields and its managed inundations play a role here is left unanswered by the data, though, as managed rice fields typically show significant jumps in seasonal backscatter time series.

## Usage Notes

The Sentinel-1 Global Backscatter Model (S1GBM) presents a new perspective on Earth’s land surface, opening up the radar vision on land-cover and land-usage. It comprises a set of globally harmonised backscatter mosaics, mapping for the period 2016–17 the land C-band microwave reflectivity in two polarisations expressed as *σ*_0_ (sigma nought) values. The mosaics are based on incidence-angle-normalised and quality-curated radar imagery observed by the Synthetic Aperture Radar (SAR) sensors onboard the Sentinel-1 satellites.

The S1GBM Version 1.0 provides at a 10 m pixel sampling two complete global land mosaics of mean backscatter normalised to an incidence angle of 38°, one for VV- and one for the VH-polarisation of the radar waves. Although similar to some extent, these two polarisation channels describe surface and vegetation properties with different sensitivity and intensity, and depict valuable input to mapping methods of land cover or usage, soil composition, geomorphology, and vegetation structure. While the VH mosaic offers higher separability of vegetation features, the VV mosaic offers in general a cleaner impression thanks to its stronger signal and less noise.

The here presented S1GBM backscatter signature analysis provides for major global land cover classes the characteristical C-band backscatter signatures of major land cover classes in VV- and VH-polarisation. (i.e. statistical values for mean and standard deviation; see Fig. 3 and Table 2).

In our chosen experiment of mapping permanent water bodies (PWB), we demonstrated the ease of integrating the S1GBM into land cover classification procedures. With the large- (Fig. 4) and small-scale examples (Fig. 2 and Fig. 5) discussed in the technical validation section, we spotlighted typical characteristics of the mosaics and illustrate the physical appearance of land surfaces in the Sentinel-1 CSAR data.

The mapping of PWB—applying a simple threshold approach to the S1GBM VV and VH mosaics—yielded very satisfying results, with almost perfect performance in biomes of the temperate and cold climates in the mid- and high-latitudes (e.g. in Switzerland, Finland, Canada). While arid and barely vegetated zones are excluded from the PWB-experiment due to our approach’s inability to differentiate robustly between dry soil and water (also owing to its simplicity), also semi-arid areas pose challenges (e.g. Great Salt Lake). Most of the disagreement between the S1GBM PWB map and the Landsat-based reference from JRC-GSW appears over areas with a high water-seasonality, i.e. areas that are covered by water during most months of the year. Our fast and simple PWB-method, applied to two years of Sentinel-1 observations reduced to single layers, not surprisingly, was not capable of correctly classifying such highly dynamic water bodies. However, particular cases of water bodies with ice-cover or emerged plants were categorised differently in the SAR and GSW maps, opening up possibilities in future water mapping endeavours. Here, we underline that the definition of water boundaries must be understood by its context, as water bodies and their outlines are differently perceived from in-situ, optical sensors, or radars (e.g. in respect to shallow or vegetated edges).

From the technical validation of the S1GBM presented here and demonstrating its use for PWB-mapping, we conclude that the overall quality of the normalised Sentinel-1 mosaics is very good. The Sentinel-1 backscatter data from the years 2016–17 is aggregated comprehensively to PLIA-normalised mosaics, albeit that the normalisation is not perfect over sparsely covered areas of the low-latitudes and some water bodies with a problematic configuration of just one or two image-geometries/orbits. The few detected remaining artefacts from the input preprocessed data within the global mosaics (e.g. Lake Albert) constitute a local degradation of the S1GBM’s quality, though we would like to stress that these were effectively marginalised through our manual quality checks during the generation of the S1GBM. In spite of the locally rather sparse Sentinel-1 coverage, the obtained mosaics of the subtropics and the tropics appears to be of excellent quality and might even outperform optical imagery, as the radar signal is undisturbed by clouds.

In conclusion, the S1GBM offers a new independent information source for the analysis of the global land surface and the inland water extent, and it provides insight into processes related to the geometric and dielectric properties of the soil and vegetation. With this, it is most suitable for supporting the design and verification of upcoming C-band radar sensors, and we are convinced it will advertise the use of radar imagery and its rich information content.

## Data Availability

The S1GBM mosaics were produced with geodata management software and scientific algorithms contained in the SAR Geophysical Retrieval Toolbox (**SGRT** v2.4) software suite, which embeds also open-access python libraries (**GDAL**^36^, **NumPy**^45^) and Sentinel-1 preprocessing functions of the **SNAP** v6.0 toolbox^30^. The SGRT suite has been developed by TU Wien and is not openly accessible, and is only available under conditions to project- and research-partners of TU Wien. For the usage of the **Equi7Grid** we provide data and tools via the openly accessible python package on GitHub^33^. Furthermore, we encourage users to use TU Wien’s open-source Python package **yeoda**, a datacube storage access layer that offers functions to read, write, search, filter, split and load data from the S1GBM datacube. The yeoda package is openly accessible on GitHub^55^.
